# Progress in Surgery of the Liver and Gall Bladder

**Published:** 1903-03-07

**Authors:** 


### Progress in Surgery of the Liver and Gall Bladder.

**Tropical Abscess of the Liver**, according to Godlee,[1] are best incised through the lower axilla in the majority of cases. If two lines be drawn vertically downwards, prolonging the anterior and posterior folds of the axilla as far as the margin of the ribs, they will at the lower part enclose the space where the widest interval intervenes between the lowest part of the pleura and the costal margin, an interval generally of 2 inches and often even greater. The incision may conveniently be made transversely—that is, parallel with the lower margin of the pleura—and the portion of one rib and cartilage that crosses the wound obliquely should be removed, great care being taken to separate the structures on the deep surface of it without injuring the pleura in case by chance it should extend lower than usual. Generally the structure thus exposed consists only of the origin of the diaphragm, but if the pleura should be low it is easily recognised, and may without any difficulty be dissected up without injuring it and fastened out of the way to the upper part of the wound. If it should accidentally have been opened, the suture of the opening is a simple matter, and it is essential to make it perfectly airtight before proceeding. The rest of the operation consists in incising the diaphragm either in the direction of its fibres (preferably) or across them, and then cutting through the diaphragmatic peritoneum. If there be no adhesions, the liver may either be sutured to the diaphragm and the chest walls, or the parts around the opening may be carefully plugged with some antiseptic material. If the latter course be adopted it must not be forgotten that if the abscess be large the liver will at once shift its position, and that this shifting will take place in the upward direction.

**Cholelithiasis.**—Ochsner[2] says that the symptoms which will most constantly lead to a correct diagnosis when gall-stones are present are not biliary colic, jaundice, and passing of gall-stones with the fæces as we have been taught for many years, but (1) digestive disturbances, a feeling of weight or burning in the vicinity of the stomach after eating, gaseous distension of the abdomen; (2) a dull pain extending to the right from the epigastric region around the right side about at a level with the tenth rib, extending to a point near the spine and progressing upward under the right shoulder-blade; (3) a point of tenderness upon pressure between the ninth costal cartilage on the right side and the umbilicus; (4) a history of having had one or more attacks of appendicitis or typhoid fever. (5) In many of these cases there is a slight tinge of yellow in the skin, not sufficient to be recognised as icterus, but still sufficient to be recognised on close inspection, especially on the days on which the patient is not feeling very well, when she complains of feeling billious. (6) There is usually an increase in the area of liver dulness. (7) There may be a swelling of variable size opposite the end of the ninth rib. In operating on the common duct Robson[3] lays stress on the employment of a firm sandbag under the back opposite to the liver, which not only pushes the spine and with it the common duct forward so that it is several inches nearer the surface, but acts like the Trendelenburg position in pelvic surgery by letting the viscera fall away from the field of operation. By lifting the lower border of the liver in bulk (if needful first drawing the organ downwards from under cover of the ribs) the whole of the gall-bladder and the cystic and common ducts are brought quite close to the surface, and as the gall-bladder is usually strong enough to bear traction, the assistant can draw it up and prevent it from falling over the common duct and so impeding the view. It will now be observed that instead of the gall-bladder and cystic duct making a considerable angle with the common duct an almost straight passage is found from the opening in the gall-bladder to the entrance of the bile duct into the duodenum, and if adhesions have been thoroughly separated, as they should always be, the surgeon has immediately under his eye the whole length of the ducts with the pancreas and duodenum. Berg[4] formulates the following conclusions: (*a*) Indications for medical treatment—cholecystitic pain or attacks of biliary colic, in either case unattended with fever; (*b*) Indications for surgical treatment. 1. Operations of choice—undertaken in the quiescent period; with the object of avoiding serious complication—a simple procedure, and followed by 2 to 3 per cent. mortality; (*a*) Severe cholecystitic pain, or oft-repeated uncomplicated attacks of biliary colic; persisting in spite of medical treatment. In virtue of which symptoms the patient becomes invalided and incapacitated for work; (*b*) After the first attack of acute cholecystitis, associated with fever. 2. Compulsory operations—difficult and laborious procedures, and attended with high mortality—50 to 75 per cent. (*a*) Foudroyant and intensely acute attacks of cholecystitis (this may be the first indication of calculous disease, but usually follows previous milder attacks); (*b*) Hydrops, empyæma, gangrene, or perforation of the gall bladder, cholæmia, abscess of the liver, and diffuse peritonitis. Lilienthal[5] enumerates the consequences of the presence of gall-stones as follows:—(1) They may lie quiescent or latent within the gall-bladder, and in this case may do little, if any, harm; (2) quiescent stones may, by constant slight irritation, cause carcinomatous degeneration of the gall-bladder or its passages; (3) gall-stones may give rise to attacks of pain of the greatest severity—even causing death—and the attacks may become so frequent as to make life practically unendurable; (4) jaundice, with all its discomforts and dangers, is a common accompaniment of the stoppage of the common duct by stones or by the swelling of the mucous membrane caused by their presence; (5) ulceration and infection of the gall-bladder; (6) great tension of the gall-bladder, often with empyæma of that organ, due to impaction of a calculus in the cystic duct; (7) gangrene and perforation of the gall-bladder, with all the dangers which these accidents imply; (8) cholangitis, extending into the finer ramifications of the biliary passages—almost uniformly fatal; (9) intestinal obstruction from adhesions around the gall-bladder, or from the presence within the gut of a gall-stone of such size as to cause obturation; (10) cirrhosis of the liver, with all its disastrous consequences from prolonged obstruction of the common duct. Moyniham[6] gives a somewhat similar list of the complications of gall-stones. Morison[7] says the best skin incision, when a difficult common-duct operation has to be done, begins one inch below the tip of the twelfth rib and ends in front in the middle line, at the upper part of the middle third of a line drawn from the ensiform cartilage to the umbilicus. Drainage is secured by leaving the opening in the duct unsutured, with a large tube apposed to it. Garrett[8] reports an unsuccessful case of operation for complete intestinal obstruction produced by a gall-stone and a band.

**Cholecystectomy** for cholelithiasis is regarded by Gibson[9] as follows:—1. In a properly selected case it is an extremely safe and simple operation. 2. It is a curative operation, doing away with subsequent attacks of cholecystitis and more remotely of renewed stone formation. 3. It eliminates the disagreeable possibilities of long-continued biliary and mucous fistulæ. 4. In certain technical conditions, such as atrophic or inaccessible bladder, obliteration of the cystic duct or impacted stone in the cystic duct, and in hemorrhagic conditions of the gall-bladder, it becomes logically the method of choice. 5. It is a prophylactic measure against the development of carcinoma on the site of long-standing irritation. 6. It offers the prospect of a shorter and easier wound-healing and convalescence. 7. It is not to be employed indiscriminately, but has its proper limitations and contra-indications. The decision to resort to it will often be determined by the existence or absence of technical difficulties attending its performance, that is, to remove it if one can do so easily and safely, otherwise simply drain (cholecystostomy). Bland Sutton[10] while recognising its usefulness and necessity under certain circumstances thinks that its high mortality makes it inferior to cholecystotomy in ordinary circumstances. In shrunken gall-bladders, in distended or inflamed gall-bladders with a stone impacted in the common duct and in cancer of the gall-bladder cholecystectomy is the best operation.

[1] Lancet, May 24, 1902, p. 1452. [2] Annals of Surgery, June 1902, p. 708. [3] Lancet, April 12, 1902, p. 1024. [4] Med. Rec., May 3, 1902, p. 687. [5] Med. Rec., May 31, 1902, p. 843. [6] Brit. Med. Journ., Nov. 8, 1902, p. 1530. [7] Brit. Med. Journ., Nov. 8, 1902, p. 1487. [8] Brit. Med. Journ., Sept. 13, 1902, p. 789. [9] Amer. Jour. Med. Sci., July, 1902, p. 119. [10] Clin. Journ., July 30, 1902, p. 232.

